# Leptospirosis as a risk factor for chronic kidney disease: A systematic review of observational studies

**DOI:** 10.1371/journal.pntd.0007458

**Published:** 2019-05-23

**Authors:** Rodrigo M. Carrillo-Larco, Carlos Altez-Fernandez, J. Gonzalo Acevedo-Rodriguez, Karol Ortiz-Acha, Cesar Ugarte-Gil

**Affiliations:** 1 Department of Epidemiology and Biostatistics, School of Public Health, Imperial College London, London, United Kingdom; 2 CRONICAS Centre of Excellence in Chronic Diseases, Universidad Peruana Cayetano Heredia, Lima, Peru; 3 Centro de Estudios de Población, Universidad Católica los Ángeles de Chimbote (ULADECH-Católica), Chimbote, Perú; 4 Facultad de Medicina Alberto Hurtado, Universidad Peruana Cayetano Heredia, Lima, Peru; 5 Instituto de Medicina Tropical Alexander von Humboldt, Universidad Peruana Cayetano Heredia, Lima, Peru; 6 Department of Clinical Research, London School of Hygiene and Tropical Medicine, London, United Kingdom; 7 Department of International Health, Johns Hopkins Bloomberg School of Public Health, Baltimore, Maryland, United States of America; Baylor College of Medicine, UNITED STATES

## Abstract

**Background:**

Leptospirosis is a worldwide prevalent zoonosis and chronic kidney disease (CKD) is a leading global disease burden. Because of pathophysiological changes in the kidney, it has been suggested that these conditions may be associated. However, the extent of this interaction has not been synthetized. We aimed to systematically review and critically appraise the evidence on the association between leptospirosis and CKD.

**Methodology/Principal findings:**

Observational studies with a control group were selected. Leptospirosis, confirmed with laboratory methods, and CKD also based on a laboratory assessment, were the exposures and outcomes of interest. The search was conducted in EMBASE, MEDLINE, Global Health, Scopus and Web of Science. Studies selected for qualitative synthesis were assessed for risk of bias following the Newcastle-Ottawa Scale. 5,981 reports were screened, and 2 (n = 3,534) were included for qualitative synthesis. The studies were conducted in Taiwan and Nicaragua; these reported cross-sectional and longitudinal estimates. In the general population, the mean estimated glomerular filtration rate (eGFR) was lower (p<0.001) in people testing positive for antileptospira antibodies (eGFR = 98.3) than in negative controls (eGFR = 100.8). Among sugarcane applicants with high creatinine, those who were seropositive had lower eGFR (mean difference: -10.08). In a prospective analysis, people with high antileptospira antibodies titer at baseline and follow-up, had worse eGFR (p<0.05).

**Conclusion:**

Although the available evidence suggests there may be a positive association between leptospirosis and CKD, whereby leptospirosis could be a risk factor for CKD, it is still premature to draw conclusions. There is an urgent need for research on this association.

## Author summary

Leptospirosis is an infection that can affect the kidneys acutely, though it seems that even after the acute infection there could be risk of a long-term impaired kidney function. The evidence on this matter is sparse and limited, thus the need to comprehensively seek, synthetize and appraise the available scientific literature. In so doing, this work has found preliminary evidence that leptospirosis may be associated with impaired kidney function as per eGFR. This work and findings strongly reveal that more research is needed to quantify and characterize the long-term risk of CKD among those who had had leptospirosis infection. The raising burden of non-communicable diseases paired with a still non-negligible burden of communicable and neglected tropical diseases in low- and middle-income countries, deserve the synergism of these two broad fields for the benefit of patients and population health.

## Introduction

Globally, the incidence, mortality and disability due to chronic kidney disease (CKD) have increased, mainly driven by established risk factors such as diabetes and hypertension.[1, 2] Despite the growing body of evidence on CKD, those cases that are not related to well-known risk factors, i.e., CKD of unknown origin (CKDu), have been less systematically studied and their risk factors have not been clearly identified.[3] A recent systematic review, which only focused on a limited geographical area (Mesoamerica) but it is the only one which has conducted a formal risk of bias assessment and meta-analysis, identified that male sex, family history of CKD and low altitude were positively associated with CKD (defined as estimated glomerular filtration rate (eGFR) <60 mL/min/1.73m^2^).[3] Moreover, the authors signalled that there was insufficient evidence to draw strong conclusions about other risk factors.[3] However, this work did not include search terms regarding infectious diseases that may be associated with CKD. This could be the case of leptospirosis,[4, 5] which impact in acute kidney injury has been well documented,[6] though has also been labelled as an emerging risk factor for CKDu.[7] Because to the best of our knowledge previous reviews have not focused on this potentially new risk factor–leptospirosis–either,[8–10] we aimed to ascertain the association between leptospirosis and CKD. We conducted a systematic review and critical appraisal of the literature. In so doing, we have summarized the available epidemiological evidence providing further insights on the strength of this emerging association and signalling potential research gaps to better understand the role of leptospirosis on CKD.

## Methods

### Protocol and registration

This is a systematic review and critical appraisal of the scientific literature. This work aimed to answer the research question: *is leptospirosis an associated factor or a risk factor for CKD*? This work followed the PRISMA guidelines (S1 Checklist) and was registered at PROSPERO (CRD42018111229).[11]

### Eligibility criteria

The population of interest included men and women of any age and geographical location; also, the study population could have included population-based samples, occupational studies or hospital-based samples. No intervention of interest was studied. The comparison group should have included people free of leptospirosis at the time of kidney assessment or at the baseline assessment for prospective longitudinal studies. The outcome of interest was reduced kidney function as per eGFR,[12] high serum creatinine or chronic kidney disease of unknown origin. Studies were selected if the exposure (independent variable) was leptospirosis diagnosis; cases should have had biological (laboratory-based) confirmation.

Studies were selected if they had followed an observational design of any kind, namely cross-sectional, case-control or cohort studies. Only original investigations were included, i.e., case reports, editorials, letters, reviews or simulation studies were excluded; in addition, any other descriptive studies where no comparison group was analysed were excluded. Finally, studies should have analysed data at the individual level, i.e., ecological studies were not included. Reports were excluded if they only studied people with established risk factors for impaired kidney health: diabetes (of any kind), hypertension and glomerulonephritis. Animal model studies were excluded as well.

### Information sources and search

The search was conducted in Ovid, including EMBASE, MEDLINE and Global Health; we also searched Scopus and Web of Science. These were searched from inception without language restrictions. The terms used in these search engines are showed in S1 Text. The search was conducted on September 27^th^, 2018.

### Study selection

Search results were downloaded, and duplicates were dropped. Titles and abstracts were screened by two independent reviewers (RMC-L, JGA-R, CA-F and KO-A) following the criteria above detailed. Results on which both reviewers agreed that should be included, as well as those results on which the reviewers disagreed, were selected for full-text examination. The full text of the selected reports was sought and studied by two independent reviewers (RMC-L, JGA-R, CA-F and KO-A) following the same criteria; if there were discrepancies between reviewers these were solved by consensus among them. Both selection phases were conducted using the online tool Rayyan.[13]

### Data collection

The reviewers developed a data extraction form which was not modified during the data collection process. This was an Excel sheet containing relevant information to answer the research question, including: ascertainment methods of the exposure and outcome of interest, levels of biomarkers of kidney function, and association estimates between leptospirosis and kidney biomarkers. A positive association between leptospirosis and CKD implied that the former was a risk factor for the latter; similarly, a negative association between leptospirosis and eGFR implied that higher leptospirosis infection (e.g., serum titers) was associated with lower eGFR thus leptospirosis was a risk factor for CKD. Data extraction was conducted by one reviewer (RMC-L) and independently verified by another reviewer (JGA-R).

### Risk of bias of individual studies

Along with data extraction, risk of bias of individual studies was assessed following the Newcastle-Ottawa Scale (NOS).[14] This process was conducted by one reviewer (CA-F) and independently verified by another reviewer (RMC-L).

### Synthesis of results

Because there were few results and large heterogeneity among them, a quantitative synthesis (e.g., meta-analysis) was not conducted. Results are summarized qualitatively, and where relevant, association estimates were summarized.

### Ethics statement

No human subjects participated in this study. Therefore, it was considered of minimal risk and no approval was sought from an ethics committee.

## Results

### Study selection

The search yielded 5,981 results (50 from Embase, Medline and Global Health; 5,556 from Scopus; and 375 from Web of Science), and after duplicates were removed 5,888 titles and abstracts were screened. After this screening process, 27 reports were studied in detail, two of which were selected for qualitative synthesis (S1 Fig). Details about the excluded studies are presented in S1 Text.

### Study characteristics

The selected studies were published in the last three years,[15, 16] and conducted in different world regions: Nicaragua (Riefkohl et al.[15]) and Taiwan (Yang et al.[16]). One report yielded cross-sectional results,[15] while the other one presented both cross-sectional and longitudinal estimates.[16] In total, these studies included 3,534 people.[15, 16] Riefkohl et al. included people based on their job (e.g., sugarcane workers or sugarcane applicants).[15] Table 1 presents additional details about the study populations.

**Table 1:** Main characteristics of selected reports.

First author	Publication year	Country	Study design	Study setting	% Women	Mean age (standard deviation)	Sample size
Yang	2015	Taiwan	Cross-sectional/Cohort	Community	51.2	Mean age = 46.6 (SD:0.7) years	3045
Riefkohl	2017	Nicaragua	Cross-sectional	Community	7	Mean age = 35.0 (SD:10.6) years	489

Both studies objectively assessed the exposure and outcome of interest using blood and urine samples (Table 2).[15, 16] Furthermore, both studies analysed more sophisticated biomarkers than creatinine alone, these included: neutrophil gelatinase-associated lipocalin (NGAL); kidney injury molecule–1 creatinine ratio (KIM–1/Cr); monocyte chemoattractant protein–1 (MCP–1); interleukin-18 (IL-18); and N-acetyl-D-Glucosaminidase (NAG).[15, 16]

**Table 2:** Exposure and outcome ascertainment in the selected reports and main results.

Study	Exposure assessment	Outcome assessment	Leptospirosis prevalence	eGFR or creatinine (e.g., mean)	Main result
Yang, 2015	Microscopic agglutination test (MAT) was used on sera; MAT for the serovar Shermani was applied and expressed as antileptospira antibody seropositivity. A cases definition included: seropositive in a MAT titer 1: 100, i.e., past exposure.	Renal function, chronic kidney disease and stages of chronic kidney disease (KDIGO 2012 criteria): microalbuminuria as an albumin-to-creatinine ratio ≥30 mg/g in first morning urine; estimated glomerular filtration rate (eGFR) based on the CKD-EPI formula. For prospective analysis additional kidney markers were studied: i) neutrophil gelatinase-associated lipocalin (NGAL) in serum and urine; ii) kidney injury molecule–1 creatinine ratio (KIM–1/Cr); and iii) monocyte chemoattractant protein–1 (MCP–1).	1034 people (of 3045) were positive for antileptospira antibody. Among the follow-up sub-sample (n = 88) as of 2011, 86.4% had been positive for antileptospira antibody at baseline.	Overall mean eGFR was 100.0 (SD: 0.4) ml/min/1.73m^2^; in people positive for antileptospira antibody mean eGFR was 98.3 (SD: 0.4) ml/min/1.73m^2^ and in those negative for antileptospira antibody was 100.8 (SD: 0.6) ml/min/1.73m^2^ (p<0.001). Regarding the sub-sample who was followed-up in 2011, people whose MAT at this time was >400 had lower eGFR than those whose MAT was between 100–200 and those with negative MAT: 92.9 (SD: 15.8), 105.9 (SD: 19.5) and 104.7 (SD: 16.7) ml/min/1.73m^2^, respectively (p = 0.039). Other assessed biomarkers did not show strong results, except KIM1/Cr ratio which was higher where MAT titer was >400 than in the other two comparison groups (MAT between 100–200 and MAT negative): 0.6 (SD: 0.3) ng/mg, 0.5 (SD: 0.3) ng/mg and 0.8 (SD: 0.3) ng/mg, respectively (p<0.05).	Following a multistage sampling design at the community level, with both cross-sectional and two-year follow-up samples, there was higher kidney injury marker (KIM1/Cr) when the antileptospira antibody MAT titer level was also high (>400). This suggests there may be a renal function decline over time associated with leptospirosis.
Riefkohl, 2017	Microscopic agglutination test (MAT) was used on serum samples with the CDC's MAT panel; seronegative was when both pre- and late-harvest titer <100. Also, Antileptospira IgM antibodies in serum were measured in all subjects with low titer seroconversion or less than 4-fold rise in MAT titer; IgM was assessed with a dipstick ELISA kit. ELISA IgM between 2 and 2.5 were borderline positive, whilst between 3 and 4 were positive. Moreover, people who had a negative IgM on the first test (pre-harvest) and a positive test at the second evaluation (post-harvest), were deemed to have had recent or current infection. Urine samples were also tested for Leptospira DNA with polymerase chain reaction (PCR) test.	Creatinine was measured in serum samples following the kinetic-rate Jaffe method; the estimated glomerular filtration rate was based on the CKD-EPI equation. The following markers of kidney function were also assessed from urine samples: creatinine, albumin, interleukin-18 (IL-18), neutrophil gelatinase-associated lipocalin (NGAL) and N-acetyl-D-Glucosaminidase (NAG). These makers would provide better evidence of kidney tubular injury hence better CKD prognosis than serum creatinine.	29.0% (MAT titer ≥100)	Among sugarcane applicants with elevated creatinine, in comparison to seronegative subjects, those seropositive had lower mean eGFR (mean difference: -10.08, 95% CI: -24.12; 3.96). During the pre-harvest phase (reference was seronegative subjects), cane cutters who were seropositive had higher IL-18 and NGAL; likewise, sugarcane applicant had higher NGAL and NAG. At the post-harvest stage, and independent of job category, seropositive sugarcane workers had higher NGAL.	

### Leptospirosis and kidney health

Yang el al. reported that 1,034 (out of 3,045) people were positive for antileptospira antibody;[16] in addition, in the follow-up subsample, 88.4% were positive at baseline. On the other hand, Riefkohl et al. reported that 29.0% of the study population had microscopic agglutination test (MAT) equal or greater than 100, i.e., suggesting a positive case of leptospirosis.[15]

In a cross-sectional analysis Yang and colleagues showed that the mean eGFR was lower in people positive for antileptospira antibodies in comparison to their negative counterparts (p<0.001): eGFR = 98.3 (SD: 0.4) ml/min/1.73m^2^ vs eGFR = 100.8 (SD: 0.6) ml/min/1.73m^2^.[16] Moreover, they reported that being seropositive for Leptospira was associated with four (univariate) and three (multivariate) less eGFR units, in comparison to those who were negative for antileptospira antibody; of note, the multivariable model accounted for seventeen potential confounders including diabetes and hypertension, established risk factors for impaired kidney health. This preliminary evidence already suggest that leptospirosis may be associated with impaired kidney function, regardless other relevant risk factors. Further analysis in people with diabetes and in individuals without diabetes showed that the effect of seropositive Leptospira was stronger among the former than the latter group.

Yang et al. also conducted a two-year follow-up finding that, among people who had antileptospira antibody titer equal or greater than 400 at both time points, i.e., at baseline and at follow-up, the eGFR was lower at follow-up (p<0.05).[16] No strong difference was retrieved in people whose titer were equal or greater than 400 at baseline and at follow-up their titer were zero or between 100 and 200.[16]

In a cross-sectional endeavour, Riefkohl’s team reported that among sugarcane applicants with elevated creatinine, those who were seropositive for antileptospira antibodies had lower mean eGFR (mean difference: -10.08, 95% CI: -24.12; 3.96) than seronegative subjects. Further details about these results, and main findings regarding other biomarkers of kidney health, are depicted in Table 2.

### Risk of bias

The risk of bias assessment is shown in Table 3, and further details are available in S1 Text. The Riefkohl’s paper was deemed to have serious risk of bias in the selection and comparability domains.

**Table 3:** Risk of bias assessment.

**STUDY**	**SELECTION**	**COMPARABILITY**	**EXPOSURE**
Riefkohl, 2017	**✵**		**✵✵**
**STUDY**	**SELECTION**	**COMPARABILITY**	**OUTCOME**
Yang, 2015	**✵✵**	**✵✵**	**✵✵✵**

The more **✵** there are, the less risk of bias.

## Discussion

### Summary of evidence

This qualitative systematic review of the literature found two observational studies addressing the association between leptospirosis and CKD.[15, 16] These reports suggest that leptospirosis may be associated with impaired kidney health, as per eGFR and other highly-sensitive kidney biomarkers (e.g., KIM–1/Cr and NGAL).[15, 16] In addition, a two-year follow-up effort including 88 individuals found that sustained high antileptospira antibodies titer was associated with worse eGFR.[16] Certain jobs may be associated with impaired kidney health, and Riefkohl et al reported lower eGFR in sugarcane applicants with positive antileptospira antibody titer.[15] The findings of this review show there is a non-negligible dearth of evidence about this association; nevertheless, after accounting for their limitations, the available evidence already suggests there may be a positive association between leptospirosis and CKD whereby leptospirosis is associated with higher odds and risk of CKD. This observation deserves further attention from the clinical and epidemiological community.

### Limitations of the review

This is a comprehensive literature review including five search engines which cover several world regions. Although our search covered relevant veterinary or zoonosis sources, we did not search any specific search engine of these disciplines which could have retrieved extra results. However, we argue this is a minor limitation because these information sources would have focused on other aspects of leptospirosis rather than on their impact on human health or clinically relevant outcomes such as kidney function.

### Limitations of the reviewed reports

Although Yang et al. studied a fairly large sample size, the two-year follow-up results only included 88 people.[16] This small sample size compromises the validity and extrapolation of their findings. In this line, the fact that Riefkohl et al.’s study population was selected based on their jobs, prevents their findings to be extrapolated to the general population too, i.e., there could have been selection bias.[15] These limitations urgently call to conduct larger and longer research efforts to better understand the true association between leptospirosis and CKD.

Even though both studies reported relevant and promising results, they did not properly account for sources of confounding bias and missing data.[15, 16] This also invites the research community to design stronger studies to address the association of interest and to analyse the results following comprehensive methods and techniques to account for missing observations and confounding factors (e.g. occupational exposure or comorbidities such as diabetes mellitus and hypertension). In addition, we invite infectious diseases researchers to also consider risk factors for non-communicable diseases when conducting research or statistical analysis.[17]

### Pathophysiology pathways between Leptospirosis and kidney disease

It has been proposed that leptospirosis may be a risk factor for CKD through two different pathophysiological pathways.[7] Acute kidney injury is a well-known complication of leptospirosis[6], which if not treated promptly could progress to CKD. Therefore, the occurrence of acute kidney injury during leptospirosis infection could signal higher CKD risk in these patients.[18, 19]

After recovery of the acute infection, some patients might persistently carry leptospirosis in the kidney, which added to other factors such as extreme heat and dehydration, could exacerbate the kidney injury and lead to CKD.[15] Animal models have shown that chronic Leptospira infection results in tubulointerstitial nephritis and interstitial fibrosis.[20] The proteins of Leptospira outer membrane provoke inflammation and tubular damage through activation of Toll-like receptors and factor-beta/Smad-associated fibrosis pathway. Toll-like receptors trigger a cascade that ends with activation of nuclear transcription factor kappa B and mitogen-activated protein kinases.[21–23] These changes would lead to irreversible kidney damage, i.e., CKD.

### Future work

To the best of our knowledge this is the first systematic review to ascertain the association between leptospirosis and CKD. Although the results support there may be a positive association between these illnesses, the epidemiological evidence is still weak and deserves additional and more comprehensive studies. Studies randomly selecting subjects from the general population, specifically in areas of high endemicity of leptospirosis, are needed to assess the strength of the association of interest. In this line, prospective cohort studies are very much needed so that preliminary evidence on causality is available. Adequate analytical methods, such as causal inference techniques, could be applied and we encourage clinicians and epidemiologist to work together on these endeavours.

From a basic science and immunology point of view, a better characterization of the involved serovars seems relevant. Yang et al. only tested for one serovar (*Leptospira santarosai* serovar *Shermani*) and found strong and even prospective evidence about a possible positive association between leptospirosis and CKD.[16] On the other hand, Riefkohl et al. tested for several serovars and found that the most common ones were *Bratislava* and *Canicola*, among others.[15] These findings may imply that the negative effect of leptospirosis on kidney function exists regardless of the serovar, at least among the ones already studied. Whether this premature conclusion is true, and whether a specific serovar has a larger effect, remains unknown.

It has been suggested that Leptospira may asymptomatically colonize the human kidney.[24] If in fact leptospirosis leads to CKD, or it is at least partially associated, new research projects could try to identify who with an asymptomatically colonization might have diminished kidney function in the future. This would also imply detecting where asymptomatically colonization is possible. This may not be a static task because due to climate change, migration or poor sanitation, one may find leptospirosis where previously there were not any cases. We encourage human and veterinary epidemiologists to work on these pending tasks.

### Conclusions

There is a serious dearth of evidence to accurately assess the association between leptospirosis and CKD. Although still premature, available observational evidence following cross-sectional and prospective designs suggest there may be an association between these conditions, whereby leptospirosis could be a potential risk factor for CKD. Given the relevance of these pathologies, leptospirosis as a worldwide-spread zoonosis and CKD as a major health and disability burden,[1, 25, 26] their association should be further studied to better understand their interaction and find new prevention avenues. The new knowledge could guide prevention strategies and explain CKD in the absence of other established risk factors.

## Supporting information

S1 Checklist PRISMA checklist. (DOCX)

S1 Fig PRISMA flow diagram. (JPG)

S1 Text Search terms and excluded studies. (DOCX)
