# Supplementary figures and images for: Leptospirosis as a risk factor for chronic kidney disease: A systematic review of observational studies
Source: PLoS Negl Trop Dis. 2019 May 23;13(5):e0007458. doi: 10.1371/journal.pntd.0007458 (PMC6550415; doi:10.1371/journal.pntd.0007458)

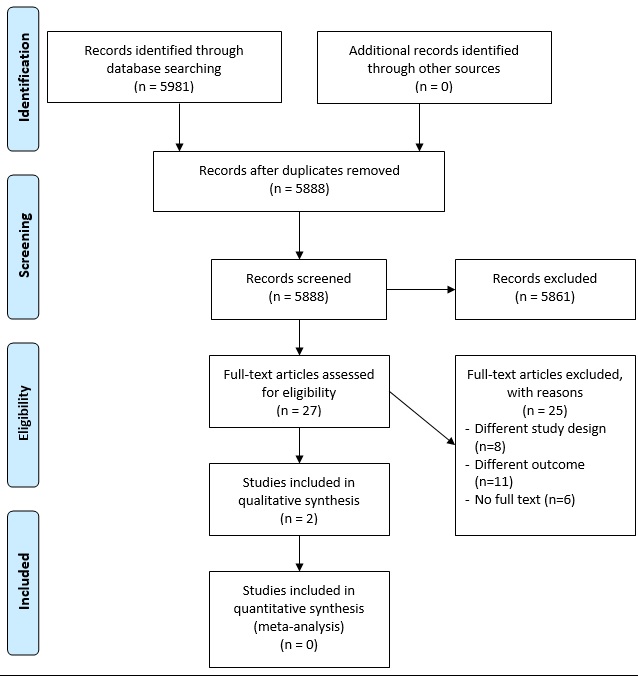

Supplement: S1 Fig — (JPG) [file pntd.0007458.s002.jpg]
