# Supplementary material for: Leptospirosis as a risk factor for chronic kidney disease: A systematic review of observational studies
Source: PLoS Negl Trop Dis. 2019 May 23;13(5):e0007458. doi: 10.1371/journal.pntd.0007458 (PMC6550415; doi:10.1371/journal.pntd.0007458)
Supplement: S1 Text — (DOCX) [file pntd.0007458.s003.docx]

**Supplementary Material**

**Leptospirosis as a risk factor for chronic kidney diseases: A systematic review of observational studies**

Rodrigo M. Carrillo-Larco

Carlos Altez-Fernandez

J. Gonzalo Acevedo-Rodriguez

Karol Ortiz-Acha

Cesar Ugarte-Gil

[SEARCH TERMS 3](#_Toc4102313)

[Search terms in Ovid, including Medline, Embase and Global Health 3](#_Toc4102314)

[Search terms used in Scopus 4](#_Toc4102315)

[Search term used in Web of science 5](#_Toc4102316)

[Excluded studies 6](#_Toc4102317)

[Risk of bias, complete assessment 27](#_Toc4102318)

SEARCH TERMS

## Search terms in Ovid, including Medline, Embase and Global Health

| 1 | (leptospirosis OR leptospira OR leptospir* OR Weil's disease OR Weils Syndrome OR Swamp Fever OR Mud fever OR Autumn fever OR Akiyami disease OR Swineherd's disease OR Rice field fever OR Cane cutter fever OR Haemorrhagic jaundice OR Hemorrhagic jaundice OR Stuttgart disease OR Canicola fever OR Fort Bragg OR icterohaemorrhagic fever OR icterohemorrhagic fever OR seven day fever OR dairy farm fever) |
| --- | --- |
| 2 | (kidney disease OR chronic kidney disease* OR chronic renal disease* OR chronic kidney failure OR chronic renal insufficiency OR ckdu) |
| 3 | 1 and 2 |
| 4 | exp animals/ not humans.sh. |
| 5 | 3 not 4 |

## Search terms used in Scopus

(ALL(leptospirosis) OR ALL(leptospira) OR ALL(leptospir$) OR ALL(Weil's disease) OR ALL(Weils Syndrome) OR ALL(Swamp Fever) OR ALL(Mud fever) OR ALL(Autumn fever) OR ALL(Akiyami disease) OR ALL(Swineherd's disease) OR ALL(Rice field fever) OR ALL(Cane cutter fever) OR ALL(Haemorrhagic jaundice) OR ALL(Hemorrhagic jaundice) OR ALL(Stuttgart disease) OR ALL(Canicola fever) OR ALL(Fort Bragg) OR ALL(icterohaemorrhagic fever) OR ALL(icterohemorrhagic fever) OR ALL(seven day fever) OR ALL(dairy farm fever)) AND (ALL(kidney disease) OR ALL(chronic kidney disease$) OR ALL(chronic renal disease$) OR ALL(chronic kidney failure) OR ALL(chronic renal insufficiency)) NOT DBCOLL(medl) AND  (LIMIT-TO ( DOCTYPE , "ar"))  AND  ( LIMIT-TO(SUBJAREA, "MEDI"))

## Search term used in Web of science

((TS=(leptospirosis) OR TS=(leptospira) OR TS=(leptospir$) OR TS=(Weil's disease) OR TS=(Weils Syndrome) OR TS=(Swamp Fever) OR TS=(Mud fever) OR TS=(Autumn fever) OR TS=(Akiyami disease) OR TS=(Swineherd's disease) OR TS=(Rice field fever) OR TS=(Cane cutter fever) OR TS=(Haemorrhagic jaundice) OR TS=(Hemorrhagic jaundice) OR TS=(Stuttgart disease) OR TS=(Canicola fever) OR TS=(Fort Bragg) OR TS=(icterohaemorrhagic fever) OR TS=(icterohemorrhagic fever) OR TS=(seven day fever) OR TS=(dairy farm fever)) AND (TS=(kidney disease) OR TS=(chronic kidney disease$) OR TS=(chronic renal disease$) OR TS=(chronic kidney failure) OR TS=(chronic renal insufficiency))) AND DOCUMENT TYPES: (Article)

# Excluded studies

| **Reference** | **Abstract** | **Reason** |
| --- | --- | --- |
| Carvajal MP, Fagerstrom KA. Epidemiology of Leptospirosis in Costa Rica 2011-2015. Current tropical medicine reports 2017;4(2):41-6. | PURPOSE OF REVIEW: Leptospirosis is a global spirochete causing chronic renal disease that is increasing in Costa Rica. This paper identifies the prevalence and risk factors of leptospirosis in Costa Rica between the years of 2011-2015., RECENT FINDINGS: Clinical cases of leptospirosis in Costa Rica demonstrated various symptoms: from asymptomatic diseases to severe cases of kidney and liver failure. A variety of diagnostic methods with varying specificities and sensitivities were employed. In Costa Rica, prevention methods such as protective clothing, decreased contact with animals, and prophylaxis of close contacts continue to be the most important factors in reducing transmission of leptospirosis., SUMMARY: In Costa Rica, the following populations should be aware of their increased risk: those living in the province of San Jose, Puntarenas, or Alajuela; being a male; being of productive years; and exposure to specific environmental factors. | Different study design (review) |
| Cetin BD, Harmankaya O, Hasman H, Gunduz A, Oktar M, Seber E. Acute renal failure: A common manifestation of leptospirosis. *Renal Failure* 2004;26(6):655-61. | Leptospirosis is an infectious disease caused by pathogenic leptospires and may vary in degree from an asymptomatic infection to severe and fatal illness. Sixteen patients (all males; aged 40 +/- 17 years) with leptospirosis were admitted to Sisli Etfal Training and Research Hospital between July 1998 and August 2003 and were retrospectively reviewed. Age, gender, occupation, clinical presentation, laboratory features, seasonal distribution of the disease, diagnostical approach, and prognostic factors were evaluated. Eleven patients were cured with no complication; four patients died of hepatic and/or renal failure. Eight patients presented with acute renal failure; seven of them needed dialytic support. One patient developed chronic renal failure and had to undergo regular hemodialysis. All deceased patients (aged 61 7 years) were anuric at admission and their serum bilirubin changed between 39-44 mg/dL (mean 41.3 +/- 2.2 mg/dL). Cured patients ranged in age from 14-62 years (34 +/- 14 years) and their serum bilirubin levels ranged from 9-35 mg/dL (23.1 +/- 11.4 mg/dL). Crystalline penicillin G 12 million U/day was administered to all patients. Six patients also received hepatic coma treatment. This study emphasizes that leptospirosis presenting with renal failure is a severe disease, and mortality is frequently related to delays in diagnosis due to lack of clinical understanding. The association of acute renal failure and jaundice should lead the clinician to suspect leptospirosis. We concluded that old age, oliguria/anuria, high serum bilirubin levels (>36 mg/dL), and high serum potassium levels might be risk factors that increase mortality in leptospirosis. | Different outcome (accurate renal failure) |
| Chagan-Yasutan H, Chen Y, Lacuesta TL, Leano PSA, Iwasaki H, Hanan F, et al. Urine Levels of Defensin alpha 1 Reflect Kidney Injury in Leptospirosis Patients. *International Journal of Molecular Sciences*;17(10). | Leptospirosis is a zoonotic disease whose severe forms are often accompanied by kidney dysfunction. In the present study, urinary markers were studied for potential prediction of disease severity. Urine samples from 135 patients with or without leptospirosis at San Lazaro Hospital, the Philippines, were analyzed. Urine levels of defensin alpha 1 (uDA1) were compared with those of neutrophil gelatinase-associated lipocalin (uNGAL) and N-acetyl-beta-D-glucosidase (uNAG). Serum creatinine (Cr) was used as a marker of kidney injury. The levels of uDA1/Cr, uNGAL/Cr, and uNAG/Cr were positive in 46%, 90%, and 80% of leptospirosis patients, and 69%, 70%, and 70% of non-leptospirosis patients, respectively. In leptospirosis patients, the correlation of uDA1/Cr, uNGAL/Cr and uNAG/Cr levels with serum Cr were r = 0.3 (p < 0.01), r = 0.29 (p < 0.01), and r = 0.02 (p = 0.81), respectively. uDA1/Cr levels were correlated with uNGAL/Cr levels (r = 0.49, p < 0.01) and uNAG/Cr levels (r = 0.47, p < 0.0001) in leptospirosis patients. These findings suggest that uDA1, uNGAL, and uNAG were elevated in leptospirosis patients and reflected various types of kidney damage. uDA1 and uNGAL can be used to track kidney injury in leptospirosis patients because of their correlation with the serum Cr level. | Different study design (basic sciences) |
| Chou L-F, Chen T-W, Yang H-Y, Chang M-Y, Hsu S-H, Tsai C-Y, et al. Murine Renal Transcriptome Profiles Upon Leptospiral Infection: Implications for Chronic Kidney Diseases. *The Journal of infectious diseases* 2018;218(9):1411-23. | Background: Leptospirosis caused by pathogenic Leptospira spp leads to kidney damage that may progress to chronic kidney disease. However, how leptospiral infections induced renal damage is unclear., Methods: We apply microarray and next-generation sequencing technologies to investigate the first murine transcriptome-wide, leptospires-mediated changes in renal gene expression to identify biological pathways associated with kidney damage., Results: Leptospiral genes were detected in renal transcriptomes of mice infected with Leptospira interrogans at day 28 postinfection, suggesting colonization of leptospires within the kidney with propensity of chronicity. Comparative differential gene expression and pathway analysis were investigated in renal transcriptomes of mice infected with pathogens and nonpathogens. Pathways analysis showed that Toll-like receptor signaling, complements activation, T-helper 1 type immune response, and T cell-mediated immunity/chemotaxis/proliferation were strongly associated with progressive tubulointerstitial damage caused by pathogenic leptospiral infection. In addition, 26 genes related with complement system, immune function, and cell-cell interactions were found to be significantly up-regulated in the L interrogans-infected renal transcriptome., Conclusions: Our results provided comprehensive knowledge regarding the host transcriptional response to leptospiral infection in murine kidneys, particularly the involvement of cell-to-cell interaction in the immune response. It would provide valuable resources to explore functional studies of chronic renal damage caused by leptospiral infection. | Different study design (basic sciences) |
| Gamage C, Damesh i, Sarathkumara YD. Chronic kidney disease of uncertain etiology in Sri Lanka: Are leptospirosis and Hantaviral infection likely causes? *Medical Hypotheses* 2016;91:16-9. | Chronic kidney disease of uncertain etiology (CKDu) has been a severe burden and a public health crisis in Sri Lanka over the past two decades. Many studies have established hypotheses to identify potential risk factors although causative agents, risk factors and etiology of this disease are still uncertain. Several studies have postulated that fungal and bacterial nephrotoxins are a possible etiological factor; however, the precise link between hypothesized risk factors and the pathogenesis of chronic kidney disease has yet to be proven in prior studies. Leptospirosis and Hantavirus infections are important zoonotic diseases that are naturally maintained and transmitted via infected rodent populations and which present similar clinical and epidemiological features. Both infections are known to be a cause of acute kidney damage that can proceed into chronic renal failure. Several studies have reported presence of both infections in Sri Lanka. Therefore, we hypothesized that pathogenic Leptospira or Hantavirus are possible causative agents of acute kidney damage which eventually progresses to chronic kidney disease in Sri Lanka. The proposed hypothesis will be evaluated by means of an observational study design. Past infection will be assessed by a cross-sectional study to detect the presence of IgG antibodies with further confirmatory testing among chronic kidney disease patients and individuals from the community in selected endemic areas compared to low prevalence areas. Identification of possible risk factors for these infections will be followed by a case-control study and causality will be further determined with a cohort study. If the current hypothesis is true, affected communities will be subjected for medical interventions related to the disease for patient management while considering supportive therapies. Furthermore and possibly enhance their preventive and control measures to improve vector control to decrease the risk of infection. | Different study design (review) |
| Herath NJ, Kularatne SAM, Weerakoon KGAD, Wazil A, Subasinghe N, Ratnatunga NVI. Long term outcome of acute kidney injury due to leptospirosis? A longitudinal study in Sri Lanka. *BMC research notes* 2014;7:398. | BACKGROUND: Leptospirosis is an important zoonotic disease of variable severity and is a common cause of acute kidney injury (AKI) in tropics. However the knowledge on long term renal outcome in leptospirosis is scarce. This study aims to assess the long-term renal outcome of AKI caused by leptospirosis., FINDINGS: Hospital records of patients who had developed AKI following leptospirosis (Serologically confirmed) presented to two Teaching Hospitals in Kandy district over 3 years from 2007 were studied. A total of 44 patients were included and they had been followed up at least for one year in out patient clinics with regular assessment including renal status. Renal histology was studied in two patients. The primary outcome measure was normalization of renal function at one year. Of the 44 patients, 31 were in the risk and injury stage (Group 1), and the rest of them were in the failure stage (Group 2) under RIFLE criteria. Of group 2 patients, 11 had abnormal renal functions on discharge. Their mean serum creatinine and GFR values on discharge were 392 mmol/l and 20 ml/min/1.73 m2. Other two patients had full renal recovery whilst in the hospital. Nine in the group 2 required renal replacement therapy by means of peritoneal dialysis, intermittent haemodialysis or haemofiltration. Seventeen out of the total had persistently abnormal renal functions on discharge. Of them 13 recovered their renal functions to normal. Four patients (9%) who belonged to group 2, had persistently abnormal renal functions after first year compatible with stage 3 chronic kidney disease (CKD). Renal histology of two patients showed tubulointerstitial lymphocyte infiltrate, tubular atrophy and interstitial fibrosis., CONCLUSION: The long term renal outcome of AKI following leptospirosis is satisfactory as only 9% of patients had abnormal renal functions compatible with early stage of CKD. Even among them, advanced CKD or dialysis dependency had not been observed. | Different outcome (acute kidney injury) |
| Mehta K, Pajai A, Bhurke S, S, e A, Bhadade R, et al. Acute Kidney Injury of Infectious Etiology in Monsoon Season: A Prospective Study Using Acute Kidney Injury Network Criteria. *Indian Journal of Nephrology*;28(2):143-52. | The epidemiological pattern of acute kidney injury (AKI) in tropical countries during monsoon reflects infectious disease as the most important cause. AKI is a confounding factor and may be overlooked by primary health-care providers and underreported in health statistics. The present study prospectively helps estimate the burden of disease and analyze etiology, clinical profile, and outcome in a tertiary care hospital of a metropolitan city in a tropical country. The study period included monsoon season of 2012 and 2013, a total of 8 months. AKI staging was done as per the AKI Network (AKIN) criteria. Patients were treated for primary disease. Renal replacement therapy (RRT) was given as required. Patients were followed up during hospitalization till recovery/death. Out of a total of 9930 admissions during this period, 1740 (17.52%) were for infections and 230 (2.31%) had AKI secondary to infectious diseases during monsoon. The incidence of AKI (230/1740) in infectious diseases during monsoon was 13.21%. The study population (n = 230) comprised 79.5% of males and the mean age was 40.95 +/- 16.55 years. Severe AKI: AKIN Stage III was seen in 48.26% of patients and AKIN Stage I in 41.74%. The most common etiology of AKI was malaria (28.3%) followed by acute gastroenteritis (23%), dengue (16.5%), leptospirosis (13%), undifferentiated fever (10.4%), more than one etiology (5.4%), and enteric fever (3.5%). RRT was required in 44.78% of patients. Requirement for RRT was maximum in patients with more than one etiology followed by leptospirosis, malaria, dengue, and least in typhoid. The overall mortality was 12.17%. In multivariate analysis, vasopressor support and assisted ventilation were risk factors for mortality. | Different outcome (acute renal failure) |
| Rajapakse S, Weeratunga P, Niloofa MJR, F, o N, Rodrigo C, et al. Clinical and laboratory associations of severity in a Sri Lankan cohort of patients with serologically confirmed leptospirosis: A prospective study. *Transactions of the Royal Society of Tropical Medicine and Hygiene* 2015;109(11):710-6. | Background: Leptospirosis results in significant morbidity and mortality. This study elucidates markers of severity in a cohort of Sri Lankan patients. Methods: Patients presenting to three healthcare institutions in the Western province of Sri Lanka with leptospirosis serological confirmed by the microscopic agglutination test (MAT) were included. Prospective data regarding demographic, clinical and laboratory parameters was extracted. Univariate associations and subsequent multivariate logistic regression models were constructed. Results: The study included 232 patients, with 68.5% (159) demonstrating severe disease. Significant associations of severe disease at a significance level of p&lt;0.05 were fever &gt;38.8°C on presentation, age &gt;40 years, muscle tenderness, tachycardia on admission, highest white cell count &gt;12 350/mm3 and &lt;7900/mm3, highest neutrophil percentage &gt;84%, haemoglobin &gt;11.2 g/dL and &lt;10.2 g/dL, packed cell volume (PCV) &gt;33.8% and &lt;29.8%, lowest platelet count &lt;63 500/mm3, highest alanine transaminase (ALT) &gt;70 IU/L and hyponatremia with sodium &lt;131mEq/L. On multivariate analysis, PCV &lt;29.8% (p=0.011; OR 3.750; CI: 1.394-10.423), ALT &gt;70 IU/L (p=0.044; OR 2.639; CI: 1.028-6.774) and hyponatremia &lt;131mEq/L (p=0.019; OR 6.413; CI: 1.353-30.388) were independent associations of severe disease. Conclusions: Severity associations were demonstrated with both clinical and laboratory parameters. There is a need for novel biomarkers for prediction of severity in leptospirosis. © The Author 2015. Published by Oxford University Press on behalf of Royal Society of Tropical Medicine and Hygiene. All rights reserved. | Different outcome (severity) |
| Luvira U, Sukahatya M, Alano F, Danguilan RA, Thang N, Lin C, et al. Clinical features of renal diseases in southeast Asia. *Nephrology*;4:S9-S11. | The renal diseases in South-East Asia are similar to other parts of the world (i.e. glomerulonephritis, diabetes mellitus, HT, obstructive uropathy, adult-onset polycystic kidney disease, nephrolithiasis and tubulointerstitial diseases). IgA nephropathy with haematuria is most common in Singapore, while IgM nephropathy with nephrotic syndrome is common in Thailand. Lupus nephritis is the most common cause of secondary glomerulonephritis and a major cause of rapidly progressive glomerulonephritis. Acute renal failure from specific infection (malaria, leptospirosis, melioidosis), from toxin exposure (snake bite, wasp sting), from exertional heat stroke, and from drugs is frequently found. Nephrolithiasis, distal renal tubular acidosis and hypokalaemia are endemic in NE Thailand. In conclusion, the broad clinical features of renal diseases in South-East Asia are similar to other regions, with additional specific causes from infections, toxic, metabolic and environmental derangements associated with these tropical locations. | Different study design (review) |
| Nair JJ, Bhat A, Prabhu MV. A Clinical Study of Acute Kidney Injury in Tropical Acute Febrile Illness. *Journal of clinical and diagnostic research : JCDR* 2016;10(8):OC01-5. | INTRODUCTION: Tropical Acute Febrile Illness (TAFI) is one of the most common causes of morbidity within the community. Acute Kidney Injury (AKI) due to infective and non infective causes is a major complication. Presence of AKI is a major cause of mortality among patients with TAFI., AIM: To study the spectrum of tropical acute febrile illness; the proportion, spectrum and staging of acute kidney injury; Renal Replacement Therapy (RRT) initiation and in-hospital mortality., MATERIALS AND METHODS: A total of 600 TAFI patients were prospectively studied at a tertiary care centre in coastal Karnataka between September 2012 and September 2014 for the aetiology of TAFI; the development and staging of AKI based on Kidney disease: Improving global outcomes (KDIGO) guidelines; the initiation of RRT and in-hospital mortality., STATISTICAL ANALYSIS: Data analysis was done using SPSS version 17.0 with statistical significance calculated using chi-square and Fisher's exact t-test for which p-value <0.05 was considered significant., RESULTS: The spectrum of TAFI, in decreasing order, was vivax malaria, leptospirosis, dengue fever, falciparum malaria, mixed malaria, enteric fever, scrub typhus and the most common aetiology was malaria. The proportion of AKI was 54%. The most common cause of AKI, its stages 2 and 3, RRT initiation and in-hospital mortality was leptospirosis; and AKI stage 1 was dengue fever. KDIGO AKI stage 1, 2 and 3 was seen in 46.9%, 31.2% and 21.9% of AKI patients, respectively. RRT initiation was required in 10.2% of AKI patients and in-hospital mortality was 3% among all patients. AKI, RRT initiationand in-hospital mortality were significantly associated with older age, fever duration and other presenting complaints, examination findings, renal function and other parameters, leptospirosis, dengue fever, falciparum malaria., CONCLUSION: The aetiology in about half of TAFI patients in coastal Karnataka was malaria. More than 50% develop AKI with greater than one-fifth of them progressing to AKI stage 3 and one-tenth requiring RRT. The most common cause of AKI, AKI stage 2, 3, RRT initiation and in-hospital mortality was leptospirosis. AKI was present in almost all patients with leptospirosis. Therefore leptospirosis was the most nephrotoxic acute febrile illness in the present study population. Dengue fever was the most common cause of AKI stage 1. Vivax malaria was the third most common cause of AKI. The factors like age, presenting complaints, examination findings, renal function and other parameters, aetiology and RRT initiation may be used to predict AKI and in-hospital mortality. | Different outcome (acute renal injurty) |
| Melnik GV, Degtyar LD. Clinicoimmunological parallels in convalescent leptospirosis patients. *Terapevticheskii Arkhiv* 2001;73(11):75-8. | Aim. To investigate clinico-immunological parallels of convalescent period in leptospiral jaundice (LJ). Material and methods. Clinical and immunological indices were studied in 121 LJ convalescents (97.6% males, age 17-45 years). Results. Acute leptospirosis period was characterized with polymorphic clinical picture impeding precise diagnosis and threatening with such complications as bacterial shock, acute renal failure, acute renal-hepatic failure, DIC-syndrome, respiratory distress. Convalescents for a long time exhibited polyorganic pathology, developed sepsis. In late convalescence one could observe affections of the heart, liver, kidneys, bones, nervous system, eyes. This can be explained by disorders in immunity, especially structural immunodeficiency, and commissures at the sites of hemorrhages. Conclusion. Polyorganic pathology in leptospirosis convalescents arises because of immunity disorders which are not associated with the disease form. In late convalescence severe complications may accompany leptospiral jaundice. | Full-text was not available |
| Maze MJ, Biggs HM, Rubach MP, Galloway RL, Cash-Goldwasser S, Allan KJ, et al. Comparison of the Estimated Incidence of Acute Leptospirosis in the Kilimanjaro Region of Tanzania between 2007–08 and 2012–14. *PLoS Neglected Tropical Diseases* 2016;10(12). | Background: The sole report of annual leptospirosis incidence in continental Africa of 75–102 cases per 100,000 population is from a study performed in August 2007 through September 2008 in the Kilimanjaro Region of Tanzania. To evaluate the stability of this estimate over time, we estimated the incidence of acute leptospirosis in Kilimanjaro Region, northern Tanzania for the time period 2012–2014. Methodology and Principal Findings: Leptospirosis cases were identified among febrile patients at two sentinel hospitals in the Kilimanjaro Region. Leptospirosis was diagnosed by serum microscopic agglutination testing using a panel of 20 Leptospira serovars belonging to 17 separate serogroups. Serum was taken at enrolment and patients were asked to return 4–6 weeks later to provide convalescent serum. Confirmed cases required a 4-fold rise in titre and probable cases required a single titre of ≥800. Findings from a healthcare utilisation survey were used to estimate multipliers to adjust for cases not seen at sentinel hospitals. We identified 19 (1.7%) confirmed or probable cases among 1,115 patients who presented with a febrile illness. Of cases, the predominant reactive serogroups were Australis 8 (42.1%), Sejroe 3 (15.8%), Grippotyphosa 2 (10.5%), Icterohaemorrhagiae 2 (10.5%), Pyrogenes 2 (10.5%), Djasiman 1 (5.3%), Tarassovi 1 (5.3%). We estimated that the annual incidence of leptospirosis was 11–18 cases per 100,000 population. This was a significantly lower incidence than 2007–08 (p<0.001). Conclusions: We estimated a much lower incidence of acute leptospirosis than previously, with a notable absence of cases due to the previously predominant serogroup Mini. Our findings indicate a dynamic epidemiology of leptospirosis in this area and highlight the value of multi-year surveillance to understand leptospirosis epidemiology. | Different outcome |
| Yang HY, Yen TH, Lin CY, Chen YC, Pan MJ, Lee CH, et al. EARLY IDENTIFICATION OF LEPTOSPIROSIS AS AN IGNORED CAUSE OF MULTIPLE ORGAN DYSFUNCTION SYNDROME. *Shock*;38(1):24-9. | Leptospirosis is the most common zoonosis in the world but remains underreported, owing to protean manifestations and ignorance about the disease among health care providers in Taiwan. From September 2000 to March 2006, surveillance of 455 patients with multiple organ dysfunction syndrome with unclear cause or clinical suspicion of leptospirosis was performed. Diagnosis was further confirmed by microscopic agglutination test or isolation of Leptospira. Cases were classified as excluded based on confirmed etiology other than leptospirosis or negative paired serologic test. Forty-two patients were confirmed as having leptospirosis, which accounted for 9.2% of total patients with multiple organ dysfunction syndrome. Forty-nine excluded cases were identified for a case-control analysis for clinical distinction. The most common presentations of leptospirosis were fever (97.6%), acute kidney injury (85.7%), and jaundice (61.9%). The leptospirosis group showed lower urine specific gravity (cutoff value, 1.0145) and enlarged kidney size (cutoff value, 11.05 cm) as compared with the excluded cases by multivariate logistics regression. Delayed antibiotic administration prolongs the duration of hospitalization (R-2 = 0.486, P < 0.01). No mortality has been found in the leptospirosis group after initiation in 2003 of rapid immunoglobulin M serology assay that showed considerably high sensitivity and specificity. Leptospirosis accounts for a salient cause of multiple organ dysfunctions in Taiwan. Early awareness of leptospirosis by distinct presentations, followed by prompt antibiotics therapy, can dramatically save the patients. The easily performed rapid immunoglobulin M serology assay is suitable as a rapid screening test for the diagnosis of leptospirosis. | Different outcome |
| Sharp TM, Rivera García B, Pérez-Padilla J, Galloway RL, Guerra M, Ryff KR, et al. Early Indicators of Fatal Leptospirosis during the 2010 Epidemic in Puerto Rico. *PLoS Neglected Tropical Diseases* 2016;10(2). | Background: Leptospirosis is a potentially fatal bacterial zoonosis that is endemic throughout the tropics and may be misdiagnosed as dengue. Delayed hospital admission of leptospirosis patients is associated with increased mortality. Methodology/Principal Findings: During a concurrent dengue/leptospirosis epidemic in Puerto Rico in 2010, suspected dengue patients that tested dengue-negative were tested for leptospirosis. Fatal and non-fatal hospitalized leptospirosis patients were matched 1:1–3 by age. Records from all medical visits were evaluated for factors associated with fatal outcome. Among 175 leptospirosis patients identified (4.7 per 100,000 residents), 26 (15%) were fatal. Most patients were older males and had illness onset during the rainy season. Fatal case patients first sought medical care earlier than non-fatal control patients (2.5 vs. 5 days post-illness onset [DPO], p < 0.01), but less frequently first sought care at a hospital (52.4% vs. 92.2%, p < 0.01). Although fatal cases were more often diagnosed with leptospirosis at first medical visit (43.9% vs. 9.6%, p = 0.01), they were admitted to the hospital no earlier than non-fatal controls (4.5 vs. 6 DPO, p = 0.31). Cases less often developed fever (p = 0.03), but more often developed jaundice, edema, leg pain, hemoptysis, and had a seizure (p ≤ 0.03). Multivariable analysis of laboratory values from first medical visit associated with fatal outcome included increased white blood cell (WBC) count with increased creatinine (p = 0.001), and decreased bicarbonate with either increased WBC count, increased creatinine, or decreased platelet count (p < 0.001). Conclusions/Significance: Patients with fatal leptospirosis sought care earlier, but were not admitted for care any earlier than non-fatal patients. Combinations of routine laboratory values predictive of fatal outcome should be considered in admission decision-making for patients with suspected leptospirosis. | Different outcome |
| Martinelli R, Luna MA, Rocha H. Is rhabdomyolysis an additional factor in the pathogenesis of acute renal failure in leptospirosis? *Revista Do Instituto De Medicina Tropical De Sao Paulo* 1994;36(2):111-4. | Leptospirosis is an important cause of acute renal failure in our environment. Although several mechanisms are implicated, the role of rhabdomyolysis in the pathogenesis of acute renal failure in leptospirosis has not been analysed. Sixteen patients with the diagnosis of leptospiroses consecutively admitted to the hospital were prospectively studied. The disease was characterized by sudden onset in all patients and, at admission, jaundice, conjunctival suffusion and myalgias. Mild to moderate proteinuria with unremarkable urinary sediment was recorded in 37.5% of the patients and abnormal levels of urea creatinine were found in 87.5% and 74.0%, respectively. Increased levels of aminotranspherase were documented in all 12 and CPK in all 10 patients studied. Serum myoglobin levels greater than 120 micrograms/l recorded in 56.2%. A correlation between myoglobin and renal failure or severity of disease, however, could not be established. | Different outcome |
| Sitprija V, Losuwanrak K, Kanjanabuch T. Leptospiral nephropathy. *Seminars in Nephrology* 2003;23(1):42-8. | Renal involvement is common in leptospirosis. Clinical manifestations vary from urinary sediment changes to acute renal failure. Renal failure is observed in 44% to 67% of patients. Hypokalemia frequently occurs. Severe hypotension is an important warning sign for the later development of renal and pulmonary complications. Prognosis of the disease is generally good except for its association with pulmonary complications, especially pulmonary hemorrhage and acute respiratory distress syndrome. Interstitial nephritis is the basic renal lesion. Vasculitis is observed in the acute phase of the disease. Tubular necrosis and interstitial nephritis are responsible for renal failure. Glomerular changes usually are not remarkable. Hemodynamic alterations, immune response, and direct nephrotoxicity are responsible for the development of renal lesions. As in many infectious diseases, decreased renal blood flow and glomerular filtration rate play a basic role. Bacterial invasion and toxicity of outer membrane with generation of cytokines, chemokines, and cellular infiltration are important in cellular injury. | Full-text was not available |
| Turhan V, Polat E, Atasoyu EM, Ozmen N, Kucukardali Y, Cavuslu S. Leptospirosis in Istanbul, Turkey: A wide spectrum in clinical course and complications. *Scandinavian Journal of Infectious Diseases*;38(10):845-52. | Patients with high fever and multiorgan involvement were investigated for the determination of frequency, clinical course and complications of leptospirosis in Istanbul. Leptospirosis was determined in 22 cases among the 35 hospitalized patients that were prediagnosed as leptospirosis according to 'Probable Leptospirosis Diagnosis and Follow-up' form. Among the leptospirosis cases 19 were male and 16 were military staff. Mean age was 35.6 y. Dark field examination (DFE), latex agglutination test (LAG), ELISA IgM, leptospirosis culture (LC) and microscopic agglutination test (MAT) were performed to confirm the diagnoses. The most frequent initial symptoms and findings were fever, fatigue, headache, nausea-vomiting and increased muscle sensitivity. Jaundice was noted only in 2 cases. A 74-y-old female patient died after the recurrence of the disease with severe rhabdomyolysis and pulmonary failure. Sagittal sinus thrombosis, perimyocarditis and chronic renal failure were major complications in another 3 patients. ELISA IgM, LC, DFE, LAG and MAT tests were positive in 68, 72, 82, 100 and 100% of the patients, respectively. As a conclusion, diagnosis of leptospirosis is usually overlooked. Clinical awareness, use of probable leptospirosis diagnosis forms and the application of different laboratory methods in the diagnosis of suspected cases may offer the chance to diagnose the leptospirosis accurately. | Different outcome |
| Lomar AV, Diament D, Torres JR. Leptospirosis in Latin America. *Infectious Disease Clinics of North America*;14(1):23. | Leptospirosis is a common disease in Latin America. Transmission to humans occurs by contact with water or soil contaminated with the urine of rodents, dogs, or livestock. Pathogenesis is still poorly understood, and bacterial toxin or virulence factors are probably responsible for many features of the disease. The anicteric form is the most frequent presentation, and its clinical picture resembles influenza or other acute febrile diseases. Ictero-hemorrhagic leptospirosis, or Well's syndrome, represents the severe form of the disease. Its clinical picture is similar to bacterial sepsis and multiple organ involvement occurs, mainly in kidneys and lungs, and causes great morbidity and mortality. Death is often related to multiple organ failure and pulmonary hemorrhages, Diagnosis is based on serology or blood, cerebrospinal fluid and urine cultures in specific media. Treatment involves a combination of antibiotics and supportive measures. | Different study design (review) |
| Yang C-W. Leptospirosis Renal Disease: Emerging Culprit of Chronic Kidney Disease Unknown Etiology. *Nephron* 2018;138(2):129-36. | Leptospirosis is the most prevalent zoonosis affecting more than 1 million populations worldwide. Interestingly, leptospirosis endemic regions coincide with chronic kidney disease (CKD) hotspots largely due to flooding and agricultural overlaps. Acute leptospirosis induces multiple organ dysfunction including acute kidney injury and may predispose to CKD and end-stage renal disease, if not treated timely. Asymptomatic infection may carry the bacteria in the kidney and CKD progresses insidiously. Histologic finding of leptospirosis renal disease includes tubulointerstitial nephritis, interstitial fibrosis, and tubular atrophy. Proximal tubule dysfunction and hypokalemia are observed in adult male workers with leptospirosis, a characteristic similarity to CKD unknown etiology (CKDu). CKDu is a form of CKD that is not attributable to traditional risk factors clustering in agricultural communities affecting young male farmers. Kidney pathology shows a chronic tubulointerstitial disease. CKDu is being reported as an endemic nephropathy across the globe. Recent surveys suggest that asymptomatic leptospira renal colonization is an overlooked risk for renal fibrosis and CKDu. Population with anti-leptospira seropositivity is associated with lower estimated glomerular filtration rate in endemic regions and carrier may progress to CKD. Leptospirosis has been considered as a risk factor for CKDu in Sri Lanka and in Mesoamerican area. Sugarcane workers in Nicaragua showed increased anti-leptospira seropositivity and higher urinary biomarkers for kidney injury. Emerging evidence with signs of infection were reported in these endemic population, indicating that leptospira exposure could play a role in CKDu as a cause of primary kidney disease or a susceptible factor when secondary injury such as heat stress or dehydration aggravates kidney disease. Therefore, leptospirosis as an emerging culprit of CKDu deserves further in-depth investigation. | Different study design (review) |
| Tunjungputri RN, Gasem MH, van der Does W, Sasongko PH, I, rio B, et al. Platelet dysfunction contributes to bleeding complications in patients with probable leptospirosis. *PLoS Neglected Tropical Diseases* 2017;11(9). | Background: Severe leptospirosis is frequently complicated by a hemorrhagic diathesis, of which the pathogenesis is still largely unknown. Thrombocytopenia is common, but often not to the degree that spontaneous bleeding is expected. We hypothesized that the hemorrhagic complications are not only related to thrombocytopenia, but also to platelet dysfunction, and that increased binding of von Willebrand factor (VWF) to platelets is involved in both platelet dysfunction and increased platelet clearance. Methodology/Principal findings: A prospective study was carried out in Semarang, Indonesia, enrolling 33 hospitalized patients with probable leptospirosis, of whom 15 developed clinical bleeding, and 25 healthy controls. Platelet activation and reactivity were determined using flow cytometry by measuring the expression of P-selectin and activation of the αIIbβ3integrin by the binding of fibrinogen in unstimulated samples and after ex vivo stimulation by the platelet agonists adenosine-diphosphate (ADP) and thrombin-receptor activating peptide (TRAP). Platelet-VWF binding, before and after VWF stimulation by ristocetin, as well as plasma levels of VWF, active VWF, the VWF-inactivating enzyme ADAMTS13, thrombin-antithrombin complexes (TAT) and P-selectin were also measured. Bleeding complications were graded using the WHO bleeding scale. Our study revealed that platelet activation, with a secondary platelet dysfunction, is a feature of patients with probable leptospirosis, especially in those with bleeding manifestations. There was a significant inverse correlation of bleeding score with TRAP-stimulated P-selectin and platelet-fibrinogen binding (R = -0.72, P = 0.003 and R = -0.66, P = 0.01, respectively) but not with platelet count. Patients with bleeding also had a significantly higher platelet-VWF binding. Platelet counts were inversely correlated with platelet-VWF binding (R = -0.74; P = 0.0009. There were no correlations between platelet-VWF binding and the degree of platelet dysfunction, suggesting that increased platelet-VWF binding does not directly interfere with the platelet αIIbβ3signaling pathway in patients with probable leptospirosis. Conclusion/Significance: Platelet dysfunction is common in probable leptospirosis patients with manifest bleeding. Increased VWF-platelet binding may contribute to the activation and clearance of platelets. | Different outcome |
| Tomaait P. Problem of the aetiology of 'endemic nephropathy'. *Lijecnicki vjesnik* 1960;82(9):701-8. | There is obviously an association between positive leptospirosis titres and ende-mic nephropathy in Croatia. On the ground of pathological changes in the kidney in cases of acute leptospirosis and in cases of endemic nephropathy, and in regard to the results of serological leptospirosis investigations, an aetiological relation-ship between past leptospirosis and endemic nephropathy seems to be likely. | Full-text was not available |
| Perez Garcia A, Tudela V, Garcia Ramos JL, Olmos M. Renal complications of leptospirosis. *Medicina do esporte* 1971;65(385):265-72. | Renal complications of leptospirosis have been studied. The patients have been divided into 2 groups. The 1st group comprises 6 patients, in the acute stage, 1 of whom developed acute renal failure with anuria of 11 days' duration, making it necessary for 4 dialyses to be performed. A study is made in the 2nd group of the repercussions, over a more or less prolonged period, on renal function of those patients who, having suffered an acute attack, were released as cured but subsequently developed interstitial nephritis leading to renal sclerosis. In this group 52 patients have been reviewed, in 2 of whom the initial cause of the nephropathy was an acute attack of leptospirosis. | Full-text was not available |
| Mel'nik GV, Degtiar LD. Renal involvement in convalescents after icterohemorrhagic leptospirosis. *Klinicheskaya Meditsina* 2000;78(12):40-3. | Icterohemorrhagic spirochetosis convalescents develop slowly regressing renal dysfunctions most frequent of which are chronic renal failure, pyelonephritis and tubulointersticial nephritis, arterial hypertension. Renal disorders may be due to immunopathological reactions followed by activation of bacterial microflora. Damage from commissures at the sites of prior hemorrhage is also possible. In bovine leptospirosis renal damage is not so severe but it tends to progression. So, renal affection in leptospirosis is a specific pathology observed in any clinical form of the disease and demands surveillance in the regions of local focuses. | Full-text was not available |
| Sukmark T, Lumlertgul N, Peerapornratana S, Khositrangsikun K, Tungsanga K, Sitprija V, et al. Thai-Lepto-on-admission probability (THAI-LEPTO) score as an early tool for initial diagnosis of leptospirosis: Result from Thai-Lepto AKI study group. *PLoS Neglected Tropical Diseases*;12(3). | Background Leptospirosis is one of the most important zoonosis in the tropics. Currently, specific laboratory diagnostic test for leptospirosis such as polymerase chain reaction (PCR) or direct culture cannot be applied at the primary care setting especially in the resource-limited countries. Therefore, clinical presentation and laboratory examination are still the primary diagnostic tools for leptospirosis. Objectives To detect clinical factors for predicting leptospirosis in suspected cases, and to create a clinical prediction score (THAI-LEPTO) that is practical and easy to use in general practice while awaiting laboratory results. Materials and methods We performed a prospective multicenter study with a development and a validation cohort of patients presenting with clinical suspicion of leptospirosis as per the WHO clinical criteria. The development cohort was conducted at 11 centers in 8 provinces around Thailand. The validation cohort was conducted at 4 centers in 1 province from the Northeastern part of Thailand. Leptospirosis confirmed cases were defined if any one of the tests were positive: microscopic agglutination test, direct culture, or PCR technique. Multivariable logistic regression was used to identify predictors of leptospirosis. The clinical prediction score was derived from the regression coefficients (original) or from the odds ratio values (simplified). We used receiver operating characteristic (ROC) curve analysis to evaluate the diagnostic ability of our score and to find the optimal cutoff values of the score. We used a validation cohort to evaluate the accuracy of our methods. Results In the development cohort, we enrolled 221 leptospirosis suspected cases and analyzed 211. Among those, 105 (50%) were leptospirosis confirmed cases. In logistic regression adjusted for age, gender, day of fever, and one clinical factor at a time, leptospirosis group had more hypotension OR = 2.76 (95% CI 1.07-7.10), jaundice OR = 3.40 (95% CI 1.48-8.44), muscle pain OR = 2.12 (95% CI 1.06-4.26), acute kidney injury (AKI) OR = 2.90 (95% CI 1.31-6.15), low hemoglobin OR = 3.48 (95% CI 1.72-7.04), and hypokalemia with hyponatremia OR = 3.56 (95% CI 1.17-10.84) than non-leptospirosis group. The abovementioned factors along with neutrophilia and pulmonary opacity were used in the development of the score. The simplified score with 7 variables was the summation of the odds ratio values as follows: hypotension 3, jaundice 2, muscle pain 2, AKI 1.5, low hemoglobin 3, hypokalemia with hyponatremia 3, and neutrophilia 1. The score showed the highest discriminatory power with area under the curve (AUC) 0.82 (95% CI 0.67-0.97) on fever day 3-4. In the validation cohort we enrolled 96 leptospirosis suspected cases and analyzed 92. Of those, 69 (75%) were leptospirosis confirmed cases. The performance of the simplified score with 7 variables at a cutoff of 4 was AUC 0.78 (95% CI 0.68-0.89); sensitivity 73.5; specificity 73.7; positive predictive value 87.8; negative predictive value 58.3. Conclusions THAI-LEPTO score is a newly developed diagnostic tool for early presumptive diagnosis of leptospirosis in patients presenting with severe clinical suspicion of the disease. The score can easily be applied at the point of care while awaiting confirmatory laboratory results. Each predictor used has been supported by evidence of clinical and pathophysiological correlation. | Different outcome |
| Raoult D, J, el P, Mailloux M, Rougier Y. Thrombocytopenia and renal failure in leptospirosis. *American Journal of Tropical Medicine and Hygiene* 1983;32(6):1464. | In a retrospective study of 60 cases of leptospirosis, the association of thrombocytopenia and acute renal failure as previously reported was confirmed. However, increased mortality, in our experience, was not associated with either thrombocytopenia or acute renal failure. | Full-text was not available |

# Risk of bias, complete assessment

**NEWCASTLE - OTTAWA QUALITY ASSESSMENT SCALE :** *Leptospira* seropositivity as a risk factor for Mesoamerican Nephropathy (Riefkohl et al.)

**Selection**

1) Is the case definition adequate?

a) yes, with independent validation **🟑**

**b) yes, eg record linkage or based on self reports**

c) no description

2) Representativeness of the cases

a) consecutive or obviously representative series of cases **🟑**

**b) potential for selection biases or not stated**

3) Selection of Controls

a) community controls **🟑**

b) hospital controls

**c) no description**

4) Definition of Controls

**a) no history of disease (endpoint)** **🟑**

b) no description of source

**Comparability**

1) Comparability of cases and controls on the basis of the design or analysis

a) study controls for _______________ (Select the most important factor.) **🟑**

b) study controls for any additional factor **🟑** (This criteria could be modified to indicate specific control for a second important factor.)

**Exposure**

1) Ascertainment of exposure

**a) secure record (eg surgical records)** **🟑**

b) structured interview where blind to case/control status **🟑**

c) interview not blinded to case/control status

d) written self report or medical record only

e) no description

2) Same method of ascertainment for cases and controls

**a) yes 🟑**

b) no

3) Non-Response rate

a) same rate for both groups **🟑**

b) non respondents described

**c) rate different and no designation**

**NEWCASTLE - OTTAWA QUALITY ASSESSMENT SCALE :** *Overlooked Risk for Chronic Kidney Disease after Leptospiral Infection: A Population-Based Survey and Epidemiological Cohort Evidence (*Yang et al.)

**Selection**

1) Representativeness of the exposed cohort

a) truly representative of the average ___ (describe) in the community **🟑**

b) somewhat representative of the average person in the community **🟑**

**c) selected group of users : PEOPLE AFFECTED BY TYPHOON**

d) no description of the derivation of the cohort

2) Selection of the non-exposed cohort

**a) drawn from the same community as the exposed cohort 🟑**

b) drawn from a different source

c) no description of the derivation of the non exposed cohort

3) Ascertainment of exposure

a) secure record (e.g., surgical records) **🟑**

**b) structured interview** **🟑**

c) written self-report

d) no description

4) Demonstration that outcome of interest was not present at start of study

a) yes **🟑**

**b) no**

**Comparability**

1) Comparability of cohorts on the basis of the design or analysis

**a) study controls for demographic characteristic (select the most important factor) 🟑**

**b) study controls for any additional factor: Medical history and Laboratory data. (These criteria could be modified to indicate specific control for a second important factor.) 🟑**

**Outcome**

1) Assessment of outcome

a) independent blind assessment **🟑**

**b) record linkage** **🟑**

c) self-report

d) no description

2) Was follow-up long enough for outcomes to occur

**a) yes (select an adequate follow up period for outcome of interest) 🟑**

b) no

3) Adequacy of follow up of cohorts

**a) complete follow up - all subjects accounted for** **🟑**

b) subjects lost to follow up unlikely to introduce bias - small number lost - > ____ % (select an adequate %) follow up, or description provided of those lost) **🟑**

c) follow up rate < ____% (select an adequate %) and no description of those lost

d) no statement
